# Postoperative analgesic effectiveness of quadratus lumborum block: systematic review and meta-analysis for adult patients undergoing hip surgery

**DOI:** 10.1186/s13018-022-03172-8

**Published:** 2022-05-19

**Authors:** Haolan Xiong, Xiaohua Chen, Wenxiu Zhu, Wuke Yang, Fuming Wang

**Affiliations:** Department of Orthopedics, Chongqing General Hospital, Chongqing, No.118, Xingguang Avenue, Liangjiang New Area, Chongqing, 401147 People’s Republic of China

**Keywords:** Quadratus lumborum block, Hip surgery, Postoperative analgesia

## Abstract

**Background:**

Quadratus lumborum block is a truncal block with several technique variations. It has been reported as providing effective analgesia for postoperative pain. The aim of this study is to determine the efficacy of the QL block in providing postoperative analgesia for hip surgery when compared with placebo or no block or other analgesic techniques.

**Methods:**

Randomized trials evaluating quadratus lumborum block benefits in elective hip surgery were sought. The primary outcome was the 24 h opioid requirement after surgery. Two independent reviewers selected the studies and extracted the data.

**Results:**

Thirteen randomized-controlled trials were included in this study. The included studies had significant heterogeneity regarding comparator groups; therefore, a limited quantitative analysis was undertaken for the comparison of QL block versus no block or placebo only. QL block reduced the opioid use by 15.78 (95% CI, 2.31 to 29.26) mg IME in the first postoperative 24 h compared with no block or placebo with no difference in static pain scores, pain grade was reduced by 2.95 (95% CI, 2.40 to 3.60) in the QL block group compared with placebo or no block in the first postoperative 24 h during movement.

**Conclusions:**

Our meta-analysis indicates that QL block may be effective for analgesia in patients after hip surgery compared with placebo or no block. There is currently limited evidence comparing QL block with other analgesic techniques for hip surgery.

**Supplementary Information:**

The online version contains Additional file available at 10.1186/s13018-022-03172-8.

## Background

Hip arthroscopy and hip arthroplasty can lead to significant postoperative pain [1]. The best treatment for early postoperative pain after hip surgery remains controversial [2]. Opioids are widely used for pain management and can cause adverse reactions, such as nausea, vomiting, dizziness and urinary retention. In contrast, regional anesthesia can well alleviate postoperative pain, avoid opioid-related side effects, and decrease the risk of developing postoperative chronic pain [3–5]. With new techniques developing rapidly, regional anesthesia has become a feasible analgesic method in more and more surgical procedures [6].

In 2007 Blanco firstly described the quadratus lumborum (QL) block [7]. Since then, several different QL blocks approaches have been developed, all of which involve LA injection at the fascia plane surrounding the QL muscle. In landmark-based technique, Jankovic et al. failed to describe the needle tip target during QL block precisely, but they found that QL block might be identical to the posterior TAP, that could be distinguished from lateral TAP by ultrasound [8]. When further studies were finished, Blanco proposed two different QL block approaches, namely QLB1 and QLB2. At almost the same time that Borglum described the transmuscular QL block, Blanco introduced ultrasound guided QL block 2 [9, 10]. These QL block techniques were described in more detail way by Elsharkawy et al. in a review, and anatomical concepts and theories about the underlying mechanisms were discussed in it [11]. Additionally, the use of intramuscular QL block have been described by some recent publications [12, 13].

A surge of new evidence has been sprung up with respect to the different kinds of QL block approaches and the effectiveness of them in postoperative analgesia of hip surgeries [14–18]. The purpose of our study is to conduct a comprehensive analysis of the relevant clinical randomized controlled trials (RCTs) to draw a conclusion of the effectiveness of the QL block in alleviating postoperative pain for hip surgery compared with sham block or other postoperative analgesia methods in patients.

## Methods and material

### Registration and protocol

We prepared this manuscript under the guide of the Preferred Reporting Items for Systematic Reviews and Meta-analysis Protocols (PRISMA-P 2015) statement guidelines [19, 20]. A predetermined protocol was used and was registered with the International Database to Register your Systematic Reviews on 15 August 2021 (INPLASY, https://inplasy.com/, INPLASY202180063).

### Study objectives

The primary outcome in this systematic review was the 24 h opioid consumption postoperatively, that was conducted between patients who had QL block and those who had ether placebo or non-block. Twenty-four hours postoperative pain grade (static and dynamic), postoperative nausea and vomiting, urinary retention, pruritus, respiratory depression, and patient satisfaction were included as secondary outcomes. We also included the measurement of analgesic efficacy and lasting time of QL block which were composed of the time to the first administration of rescue analgesic drug and the pain grade at several time points.

Firstly, we compared QL block with sham block or no block, then the comparison that we carried out was between postoperative outcomes of QL block with other forms of regional anesthesia, such as iliac fascia block, and other regional nerve blocks. If possible, subgroup analysis stratified by QLB approach or type of surgery would be conducted.

### Search strategy

An electronic search strategy was designed which combined keywords: “joint replacement,” “joint arthroplasty,” “hip replacement,” “hip arthroplasty,” “TJR,” “TJA,” “THR,” “THA,” and “quadratus lumborum.” We finished searches of PubMed, EMBASE, Google Scholar, clinicaltrials.gov register, and Web of Science citation index. Two authors conducted all searches independently and after the search process discrepancies were discussed. We included studies written in English and Chinese. Retrospective studies, case reports, and studies where catheter techniques were used were excluded.

### Study selection criteria

Two authors (XH and CX) independently conducted literature search and screening, and when the search was finished disagreement was discussed, and when there was a disagreement, it was settled by WF. Using the following criteria, trials were firstly selected based on the title and abstract. Randomized controlled trials that conducted the comparison of the effects and outcomes of single injection QL block with placebo or other regional analgesic technique (e.g. Fascia iliaca block) in adult patients were included. Studies with incomplete clinical trials, patients under 18-year-old, or non-RCT studies were excluded.

### Data extraction

Two reviewers independently extracted the data from the included studies. At first, the characteristics including titles, authors, year of publication, study design, description of control and intervention, and number of included patients of the included studies would be summarized. Then, time to first administration of rescue analgesia, pain scores and opioid consumption at the time points mentioned above, and risk of postoperative nausea and vomiting (PONV), or other opioid-related complications were extracted. All opioid analgesics were transformed to intravenous morphine equivalents (IME) based on a standard conversion table [21]. Finally, the disagreements of the extracted data were resolved through discussion.

Two authors independently assessed risk of bias, and when there were any disagreements, they would be settled by WF, based on the Cochrane Collaboration tool for assessing risk of bias [22, 23]. The assessment of the studies included randomization, allocation concealment, participants and personnel blinding, observer blinding, incomplete data and selective reporting; each category of the study was assigned “low risk”, “high risk”, or “unclear risk”.

### Statistical analysis

We performed the meta-analysis of outcomes reported in above two studies, and we reported results in a descriptive manner if only one or two studies were available. Review Manager V5.3. was used to analyze the data. We calculated heterogeneity (*I*^2^) for each analysis result and defined the *I*^2^ statistic of 25–50% as low, the *I*^2^ statistic of 50–75% as moderate, and ≥ 75% as high [24]. If there was low heterogeneity, we chose the fixed-effect model to show the best estimate of the intervention effect. If there was moderate or high heterogeneity, the effect of the intervention was assumed to be different in each included study but conformed to the same distribution, and the random-effects model was selected to show the average intervention effect. Continuous homogenous results were combined using mean differences and reported as mean differences of 95% confidence intervals (CIs). We converted various opioids into intravenous morphine equivalents for comparison between the different trials. Dichotomous outcomes were reported as odds ratio with 95% CIs.

## Result

### Search results

**Fig. 1:**
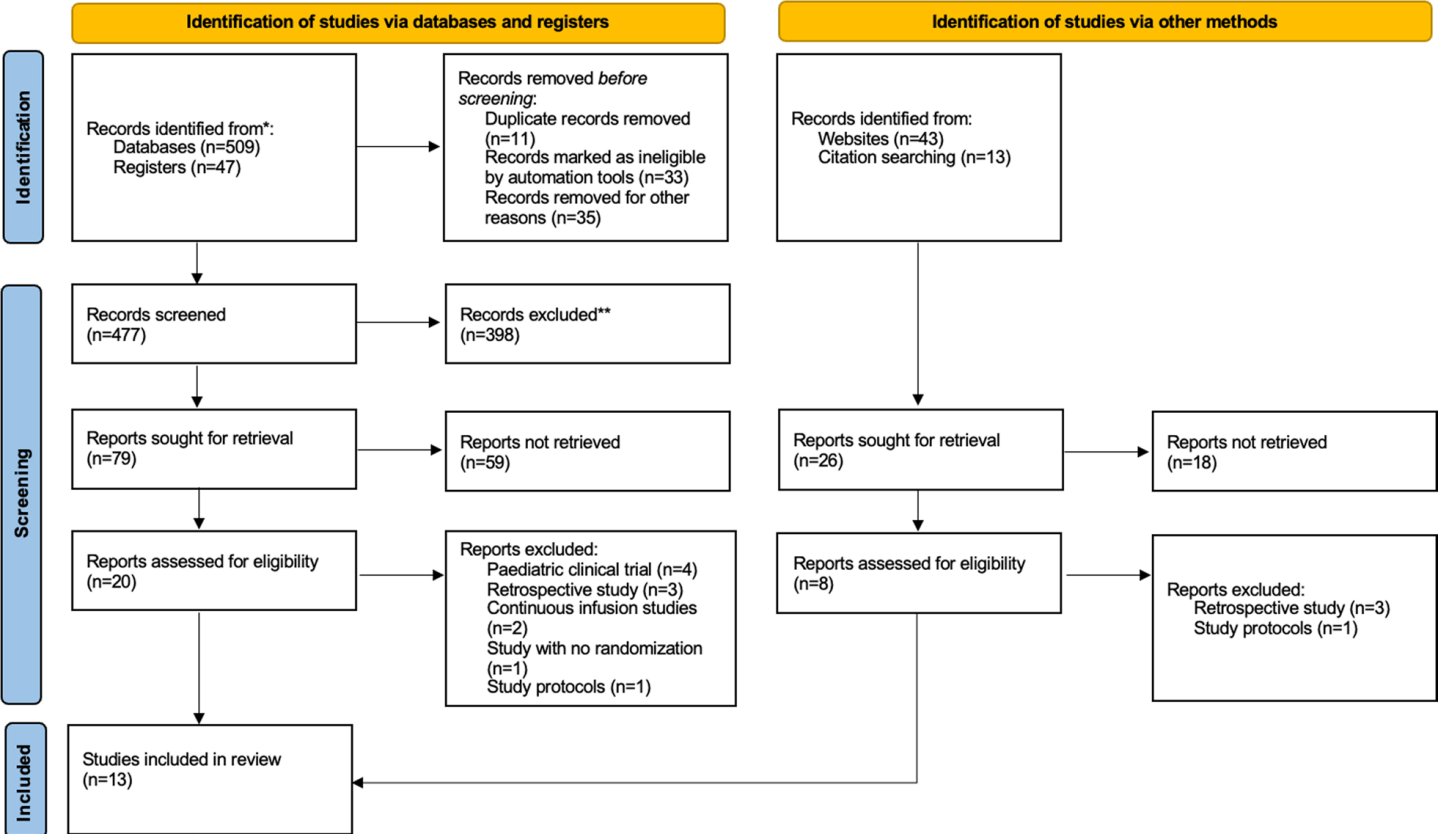
Flow chart of literature search

**Fig. 2:**
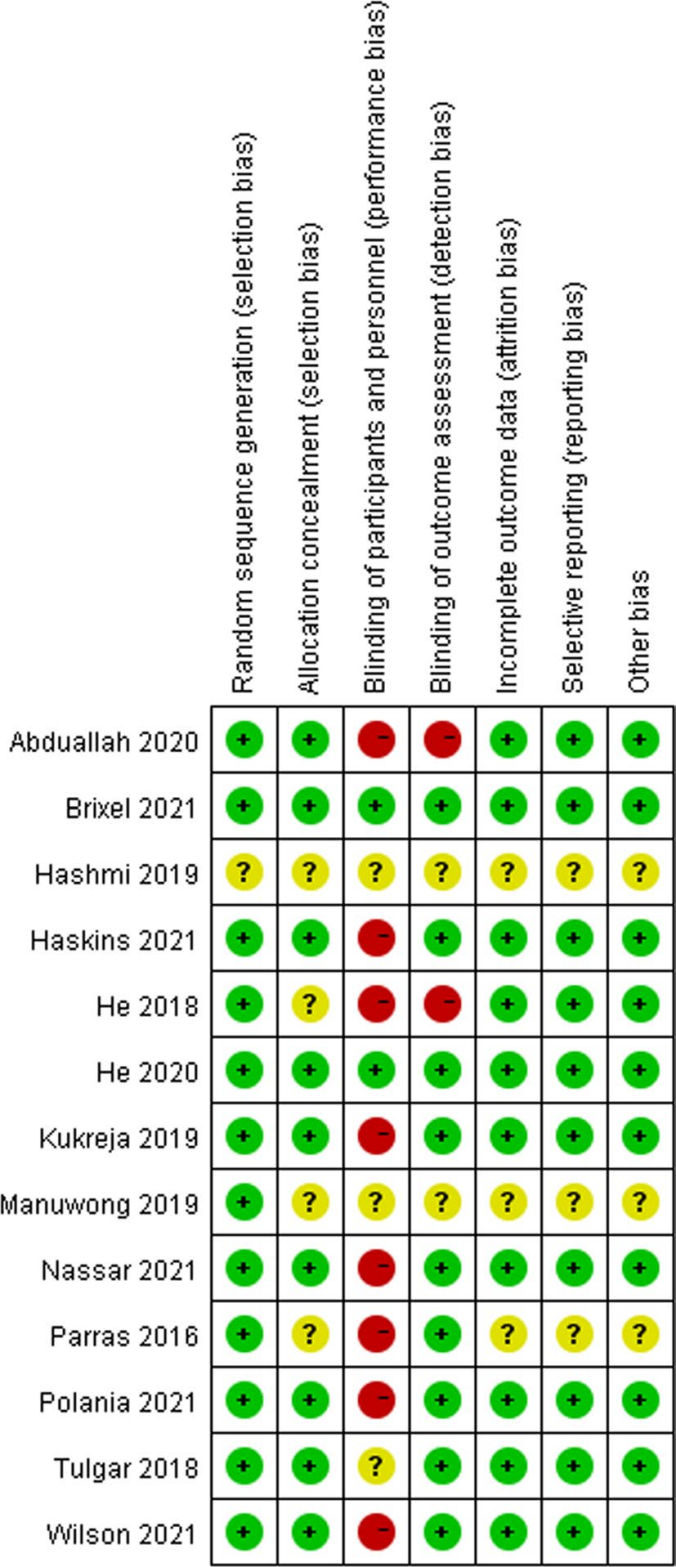
Risk of bias assessment according to Cochrane Collaboration tool for assessing risk of bias

After the initial database search, 612 citations were found out. The flow diagram is presented in Fig. 1 and Additional file 1, and the PRISMA checklist was presented in Additional file 2. We included 13 studies (11 full reports and 2 abstracts) after deleting duplicates [14–18, 25–32]. The final included trials were finished between 2016 and 2021. And Table 1 shows the participants, interventions, comparators, and summary of main findings of all the trials included in this study. The risk of bias in all aspects for every study included is shown in Fig. 2. The main sources of bias were the blinding of the outcome assessment, the lack of description for the allocation concealment, and the blinding of the included patients.

**Table 1:** Characteristics of included studies

	Methods	Participants	Intervention	Comparator	Summary of main findings
Brixel [14]	RCT	100 patients for total hip arthroplasty under general anesthesia	PQL block versus saline	Morphine requirement Pain score at rest and on movement PONV, urinary retention, hospital length of stay	No difference in morphine requirement, pain score, PONV, urinary retention and length of stay
He [15]	RCT	60 patients for total hip arthroplasty under spinal anesthesia	TQL block versus no block	Sufentanyl requirement Pain score at rest and on movement PONV, pruritis Patient satisfaction	Lower sufentanyl requirement Lower pain score Less PONV and pruritus Better satisfaction
Abduallah [26]	RCT	60 patients for total hip arthroplasty under spinal anesthesia	TQL block versus saline	Morphine requirement Pain score at rest PONV, motor block, time to the first analgesia request, patient satisfaction	Lower morphine requirement Prolongation of the time to the first analgesia request Lower VAS scores No difference in patient satisfaction
He [27]	RCT	88 patients for total hip arthroplasty under spinal anesthesia	TQL block versus saline	Morphine requirement Pain score at rest and on movement PONV, urinary retention The 10-m walk test Patient satisfaction	Lower morphine requirement Lower pain score Higher 10-m walking speed Lower incidences of nausea, vomiting, and urinary retention Better satisfaction
Kukreja [28]	RCT	80 patients for total hip arthroplasty under spinal anesthesia	TQL block versus no block	Morphine requirement Pain scores at rest Patient satisfaction, hospital length of stay, time to first opioid request	Lower morphine requirement Lower pain scores Better satisfaction No difference in length of hospital stay and time to first opioid request
Haskins [18]	RCT	96 patients for hip arthroscopy under spinal anesthesia	TQL block versus no block	Morphine requirement Pain score at rest and on movement PONV, urinary retention, hypotension Patient satisfaction	No difference in morphine requirement, pain score, satisfaction and the incidence of PONV, urinary retention, and hypotension
Tulgar [16]	RCT	60 patients for hip and proximal femur surgery under general anesthesia	TQL block versus erector spinae block versus no block	Tramadol requirement Pain score at rest	QLB and ESB both lower tramadol requirement and lower pain score No difference in PONV
Wilson [30]	RCT	46 patients for hip arthroscopy under general anesthesia	LQL block versus saline	Morphine requirement Pain score at rest and on movement Patient satisfaction	No difference in morphine requirement, pain score, satisfaction and the incidence of side effects
Manuwong [32]	RCT-abstract	40 patients for total hip arthroplasty under spinal anesthesia	TQL block versus no block	Morphine requirement Pain score at rest and on movement Time to first step, adverse effects Patient satisfaction	No difference in morphine consumption, pain score, time to first step, adverse effects and patient satisfaction
Nassar [25]	RCT	36 patients for hip arthroplasty (both total hip arthroplasty and hip hemiarthroplasty) under spinal anesthesia	TQL block versus Fascia Iliaca	Morphine requirement Pain score at rest and on movement Postoperative quadriceps muscle power	FIB showed lower 24 h morphine consumption, QLB showed better quadriceps motor power No difference in pain score
Hashmi [29]	RCT-abstract	48 patients for total hip arthroplasty under spinal anesthesia	TQL block versus fascia iliaca block	Opioid requirement Pain score at rest Motor block	No difference in opioid requirement and pain score at rest, and motor block
Parras [31]	RCT	97 patients for hip hemiarthroplasty under spinal anesthesia	LQL block versus femoral nerve block	Opioid requirement Pain score	Lower morphine requirement and pain score
Polania [17]	RCT	46 patients for total hip arthroplasty under spinal anesthesia	TQL block versus lumbar plexus block	Morphine requirement Pain score at rest and on movement Time to achieve 100 feet of walking	No difference in pain score, opioid consumption and time to achieve 100 feet of walking

*PONV* postoperative nausea and vomiting; *QL* quadratus lumborum; *LQL* lateral quadratus lumborum; *PQLB* posterior quadratus lumborum; *TQLB* transmuscular quadratus; *FIB* fascia lilac block

According to the anatomical site of local anesthetic (LA) deposition, 3 major anatomic variants of QL block have been described. The names of different QL block approaches are inconsistent in the literature. In this study, we used the anatomical technical terms lateral, posterior, and transmuscular QL block. Lateral QL block, namely ‘‘QLB-1’’, involves injection of local anesthetic at the anterolateral aspect of the QL muscle. Posterior QL block, namely ‘‘QLB-2’’, involves injection of local anesthetic at the posterior border of the QL muscle. Transmuscular QL block, namely ‘‘anterior QL block’’ or ‘‘QLB-3’’, involves LA injection between the anterior border of the QL muscle and the anterior thoracoabdominal fascia.

The type, concentration, and dose of local anesthetic vary from trial to trial. Regarding dose, 11 trials [14–18, 25, 26, 28–30] used a pre-determined volume of LA, with each injection of 20 to 40 ml. One of the studies [27] included used a dosage regimen of 30 ml of 0.33% ropivacaine in patients with a body weight > 75 kg, or 25 ml 0.33% ropivacaine in patients with a body weight of 50–75 kg, or 20 ml 0.33% ropivacaine in patients with a body weight of 30–50 kg, respectively. The LA dosage regimen was not described in one of the abstracts [32]. Regarding the LA type, 5 trials [14, 15, 17, 27, 30] used ropivacaine (0.25–0.5%) and 6 [18, 25, 26, 28, 29, 32] used bupivacaine (0.25%). One trial [16] used a mixture of LA (bupivacaine with lidocaine), and one trial [31] used levobupivacaine (0.125, 0.25%).

All the included studies involved adult patients who underwent hip surgeries. The types of surgeries are listed in the Table 1. The included studies used QL block for the surgeries as follows: hip arthroplasty (10 studies [14, 15, 17, 25–29, 31, 32]), hip arthroscopy (2 studies [18, 30]) or hip and proximal femur surgery (1 study [16]). Among those ten studies applying QL block in hip arthroplasty, eight [14, 15, 17, 26–29, 32] utilized QL block only for total hip arthroplasty, one [31] used it for hip hemiarthroplasty alone and another one [25] employed it for both total hip arthroplasty and hip hemiarthroplasty.

The included trials all compared one specific QL block with either placebo (sham block)/no block, or another analgesic technique. The comparators used were placebo (sham block) [14, 26, 27, 30], no block [15, 16, 18, 28, 32], other regional anesthesia techniques (femoral nerve block [31], fascia iliaca block [25, 29], lumbar plexus block [17], and lumbar erector spinae plane block [16]). Because of the small number of studies, subgroup analysis stratified by QLB approach was impossible.

### QL block versus placebo or no block

There was significant heterogeneity in the comparators, and outcomes measured between the studies included in this review. Therefore, we only conducted the planned meta-analysis for QL block versus sham block or non-block for patients undergoing hip surgery. Table 1 demonstrates the main results of each study.

#### Primary outcome: opioid consumption in the first postoperative 24 h

**Fig. 3:**
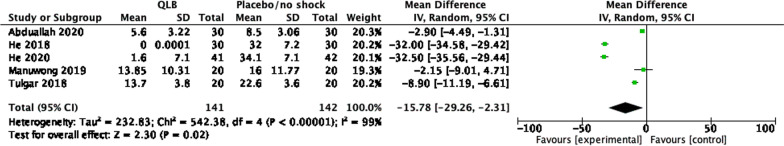
Forest plot comparing the 24 h opioid requirement of QL block group and  sham block or no block

There were nine studies [14–16, 18, 26–28, 30, 32] comparing QL block (any approach) with placebo or no block. Of these, the data from 5 studies [15, 16, 26, 27, 32] with 283 patients were presented as mean ± SD and were included in our review. The pooled estimates from these studies indicated that opioid requirement was decreased by 15.78 mg (95% CI, 2.31 to 29.26) in the QL block group compared with sham block or no block in the first postoperative 24 h (Fig. 3). Based on the surgery type, one study on hip and proximal femur surgery [16] was eliminated, and then meta-analysis of RCTs [15, 26, 27, 32] reporting only total hip arthroplasty results revealed no significant difference in opioid consumption between QL block and sham block or no block groups (mean difference − 17.48, 95% CI − 35.89 to 0.93, *I*^2^ = 99%).

#### Secondary outcome: pain scores

**Fig. 4:**
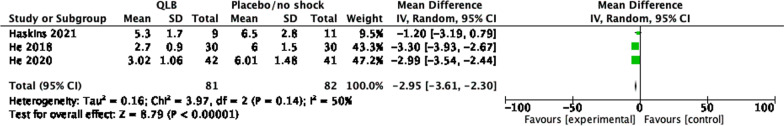
Forest plot comparing the pain grade of QL block group and  sham block or no block during movement

Nine studies presented the static pain scores at 24 h postoperatively [14–16, 18, 26–28, 30, 32]. Of these, the data of 4 studies [15, 16, 18, 27] with 203 patients were available as mean ± SD and these results were included in the meta-analysis. And there was no significant difference in postoperative static pain grades at 24 h postoperatively between the QL block and the comparators: mean difference − 0.76 (95% CI − 1.62 to 0.10), *I*^2^ = 93%. After excluding one study on hip arthroscopy [18] and one regarding hip and proximal femur surgery [16], the data of only two studies were available [15, 27], making it impossible to carry out subgroup analysis. Based on the meta-analysis of static pain scores at 12 h postoperatively, according to three studies [15, 16, 27] with 183 patients, the result was the same with the results of static pain scores at 24 h after the surgery: [mean difference − 1.24 (95% CI − 2.73 to 0.24), *I*^2^ = 92%]. There were three studies [15, 18, 27] which reported pain grade at 24 h postoperatively during movement. The pooled estimates from these studies showed that pain grade was reduced by 2.95 (95% CI, 2.30 to 3.61) in the QL block group compared with sham block or non-block group in the first postoperative 24 h on movement (Fig. 4). As only data from three studies were available, it was impossible for subgroup analysis of RCTs stratified by the surgery type.

#### Secondary outcome: opioid-related complications

**Fig. 5:**
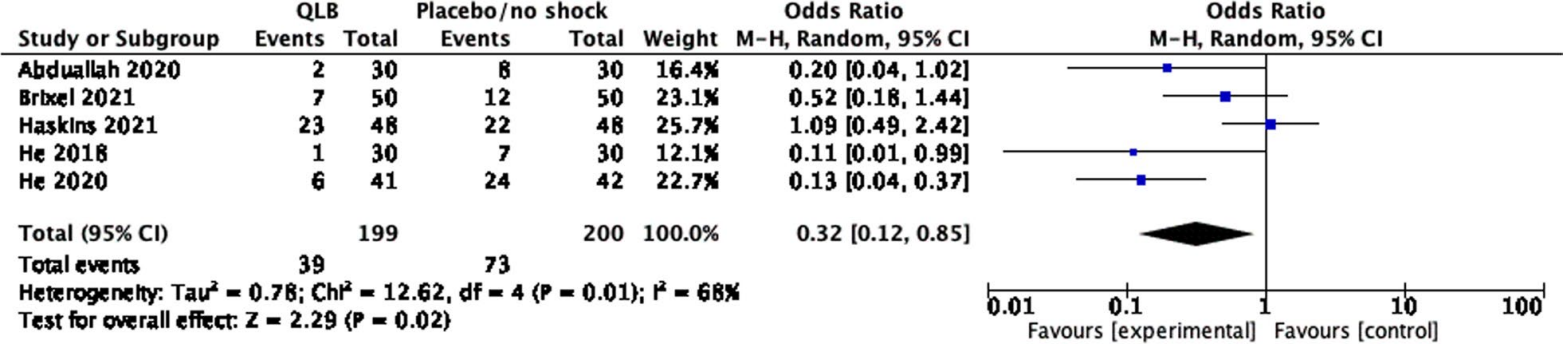
Forest plot comparing the PONV incidence of QL block and sham block or no block

The incidence of PONV was reported in 6 studies [14–16, 18, 26, 27] of which 5 [14, 15, 18, 26, 27], with 399 participants, were included in the meta-analysis. The evaluation time points varied widely or are not described. Overall, QL block decreased the incidence of PONV: odds ratio (OR) 0.32 (95% CI, 0.12 to 0.85), *I*^2^ = 68% (Fig. 5). Based on the type of surgery, one study on hip arthroscopy [18] was excluded, and the result remained unchanged, revealing that QL block decreased the incidence of PONV (OR 0.23, 95% CI 0.10 to 0.49, *I*^2^ = 24%).

Four studies [14, 15, 18, 27] reported the risk of urinary retention, of which 3 [15, 18, 27], with 339 participants, were included in the meta-analysis. Meta-analysis demonstrated significantly lower incidence of urinary retention in the QL block cohort: odds ratio (OR) 0.42 (95% CI, 0.19 to 0.95), *I*^2^ = 0. Subgroup analysis of RCTs stratified by the surgery type was not conducted since just data from three studies were available.

The incidence of pruritus was reported in 3 trials [15, 26, 27] with 202 patients, that were included in the meta-analysis. Overall, there was no difference in the incidence of prutitus between the QL block and the comparators: odds ratio (OR) 0.43 (95% CI 0.17 to 1.10), *I*^2^ = 39%. Subgroup analysis of RCTs stratified by the surgery type was not performed due to the small sample size (*n* = 3 studies).

#### Other outcomes and side-effects

Other outcomes reported (such as the rates of rescue analgesia, patient satisfaction) varied widely. He et al. [15] showed that when compared with non-block group, the incidence of administration of rescue analgesic drugs in QLB group were significantly reduced (χ (2) = 49.091, 42.857, all *P* < 0.01) and the overall satisfaction scores in QL block group were significantly higher (*t* = 7.841, *P* < 0.01). Abduallah et al. [26] reported that compared with the control group, the use of QLB in the second group significantly prolonged the time to the first need for analgesia (*P* < 0.0001). Kukreja et al. [28] showed a higher mean (standard error [SE]) patient satisfaction score (9.14 (0.28) vs. 7.46 (0.41) in the QL block group. Wilson et al. [30] reported that three patients in the placebo group (12.5%) needed a rescue block in PACU for intolerant pain despite of the use of systemic analgesics.

The rates of related adverse reactions such as hypotension, respiratory depression, or motor weakness were relatively low. Brixel et al. reported motor weakness in one patient in each group [14]. He et al. reported respiratory depression in one patient in the no block group [15]. Abduallah et al. reported side effects (bradycardia in seven, hypotension in four and hematoma in three patients) in the QL block group [26].

### QL block versus fascia lliac block

Only two studies compared QL block with fascia Iliaca block. Because of the limited number of related studies, meta-analysis was not performed. Therefore, we just described the findings of these studies. Nassar et al. [25] indicated that no significant difference in postoperative visual analog scale was found, but fascia Iliaca block showed slightly lower 24 h morphine requirement and QL block showed better quadriceps motor power. Hashmi et al. [29] found that QL block did not show better analgesia or reduced motor block than fascia iliac block in patients undergoing hip replacement surgery.

### QL block versus femoral block

Only 1 trial compared QL block with femoral block, and indicated that compared with femoral nerve block, lateral QL block reduced mean opioid requirement [9.7 (7.0) vs. 17.0 (11.2) mg IME] and VAS score at 6, 12, 18 and 24 h postoperatively (*p* < 0.01) [31].

### QL block versus lumbar plexus block

Polania et al. included 46 patients who underwent hip arthroplasty and found that there was no significant difference between the QL block and lumbar plexus block on the postoperative pain scores and total opioid consumption in the first 24 h after the surgery [17].

### QL block versus erector spinae block

Only one study compared QL block with erector spinae block. Tulgar et al. [16] compared transmuscular QLB with erector spinae block for hip and proximal femur Surgery and found that there were no differences in Numeric Rating Scale (NRS) scores and total tramadol consumption at any time points between the block groups.

## Discussion

In this systematic review and meta-analysis, thirteen clinical trials regarding QL block were identified, including nine that compared QL block with placebo or no block. This review suggested that, for patients undergoing hip surgery, QL block significantly reduced opioid consumption compared with sham block.

However, different surgery types, including total hip arthroplasty, hip hemiarthroplasty, hip arthroscopy, and hip and proximal femur surgery, were included in this study. The severity of pain might vary according to the type of surgery performed, which might contribute to opioid consumption and pain score. Subgroup analysis stratified by the surgery type was conducted in our study if possible. After excluding one study on hip and proximal femur surgery [16], the meta-analysis results of RCTs [15, 26, 27, 32] reporting only total hip arthroplasty revealed no significant difference in opioid consumption between QL block and sham block or no block groups. Considering the high heterogeneity in quantitative analysis (99%), we tend to make cautious conclusions for the effectiveness of QL block on one specific type of surgery. However, as for pain scores, subgroup analysis stratified by the surgery type was not performed, since only two or three studies were available.

In addition, this review did not note any serious complications in studies reporting opioid or block-related adverse outcomes. QL block dramatically reduced the incidence of PONV. In line with the surgery type, one study on hip arthroscopy [18] was excluded, and the result remained unchanged. As demonstrated by meta-analysis, the incidence of urinary retention significantly decreased in QL block cohort. Further, there were no significant differences in the incidence of pruritus between QL block and placebo or no block groups. Other complications included motor weakness, bradycardia, hypotension, and hematoma. Motor weakness was reported in one patient from each group in the study by Brixel et al. [14]. In the study by Abduallah et al. [26], side effects were reported in QL block group, including bradycardia (*n* = 7), hypotension (*n* = 4) and hematoma (*n* = 3). He et al. [15] found that compared with no block group, the QL block group had significantly decreased rates of administration of rescue analgesic medication to relieve pain and remarkably increased overall satisfaction scores. In Abduallah et al.’s study [26], compared with control group, the use of QL block in the second group significantly prolonged the time to the first call for analgesia. Kukreja et al. [28] reported a higher mean patient satisfaction score in QL block group. In Wilson et al.’s study [30], 3 patients in the placebo group (12.5%) required a rescue block in PACU for the intolerant pain, even though systemic analgesics were applied. In our systematic review, some studies compared QL block with other analgesic techniques. However, due to the limited existing evidence, no conclusions could be drawn. Nassar et al. [25] found no significant difference in the visual analog scale (VAS, static and dynamic) after the surgery, but fascia iliac block led to the slightly lower 24-h morphine consumption, while QL block exhibited the superior quadriceps motor power. Hashmi et al. [29] discovered that QL block did not provide superior analgesia or inferior motor block to fascia iliac block in patients undergoing hip replacement surgery. Moreover, Parras et al. [31] compared lateral QL block with femoral nerve block for hip hemi-arthroplasty. According to their results, QL block group had lower mean (SD) opioid requirement [9.7 (7.0) vs. 17.0 (11.2) mg IME] and VAS score at 6, 12, 18 and 24 h (*p* < 0.01). As found by Polania et al. [17], compared with lumbar plexus block, QL block did not cross the non-inferiority delta of two points on the NRS pain scores, and differences in total opioid consumption at 24 h were not significant. Tulgar et al. [16] compared transmuscular QL block with erector spinae block and discovered no difference in NRS score or total tramadol consumption at any time point between the block groups.

It is still controversial about whether QL block can be safely performed in the case of coagulopathy or in the anti-coagulated patient [33]. Some practitioners suggest that plane blocks may be safe with changes in coagulation function [34]. As warned by the latest evidence-based guidelines for regional anesthesia use in patients receiving antithrombotic or thrombolytic therapy released by the American Society of Regional Anesthesia and Pain Medicine, deep regional anesthesia performed in the anti-coagulant patient may result in significant morbidity that has already been reported in multiple case reports [35]. Ten of our included studies [14–18, 25–28, 31] set coagulopathy and/or therapeutic anticoagulation and/or contraindication to spinal anesthesia as the exclusion criteria, and only three [29, 30, 32] did not mention the relevant exclusion criteria.

Although all the QL block methods involve the deposition of LA around the QL muscles, each of them may have different efficacy or benefits. In our systematic review, all types of QL block achieved beneficial effects. Unfortunately, there were few studies comparing different QL block approaches. However, in a recently published study, similar postoperative tramadol consumption levels and VAS scores were identified between lateral QL block (QLB1) and posterior QL block (QLB2) [36]. In contrast, Wei et al. [37] reported that, compared with posterior TAP block (known as QLB1 placement), posterior QL block (QLB2) significantly reduced the postoperative sufentanil consumption after laparoscopic colorectal surgery.

Certain limitations should be noted in this meta-analysis, mainly including the heterogeneity in our results. There was a high heterogeneity level in our primary outcome analyses, which might be explained in several aspects. Firstly, the surgery type in the included studies varied from total hip arthroplasty, hip hemiarthroplasty, hip arthroscopy, to hip and proximal femur surgery. Therefore, the severity of pain might be different according to the type of surgery performed, and this might affect the opioid consumption and pain score. This work attempted to carry out subgroup analysis stratified by the surgery type if possible. However, sometimes subgroup analysis was impossible since only two or three studies were available. Secondly, the QL block approach varied from one study to another. Because of the small number of studies, subgroup analysis stratified by QLB approach was impossible. Besides, the control groups in the included trials were also different, including sham block, no block, femoral nerve block, fascia iliaca block, lumbar plexus block, and lumbar erector spinae plane block. Thirdly, some studies only included limited number of patients, putting them at risk of overestimating therapeutic effect. Fourthly, due to the difficulties in blinding block techniques, some of the included studies had a medium–high risk of bias. Fifthly, different concentrations and volumes of local anesthetics might affect the analgesic effect obtained. Sixthly, some studies were published in the abstract form or presented unusable data. Finally, the use of additional analgesics, such as NSAIDs or acetaminophen, was not considered in the analysis, since subgroup analysis was impossible because of the varying analgesic drugs used after operation and the small sample size.

As the breadth of evidence increases, future studies with large sample sizes and standardized endpoints will be required to evaluate the analgesic effectiveness of QL block after hip surgery. Then, the results which were measured on the same scale can be pooled and analyzed to conclude the effectiveness of QL block. Safety issues should be addressed as they may limit the use of QL block, especially in anti-coagulant patients. Meanwhile, longer-acting local anesthetics such as liposomal bupivacaine can be used to prolong the blocking effects of QL block. In addition, more studies are warranted to compare QL block with other analgesic methods. Some study [38] has already adopted the continuous catheter techniques, but more studies should be performed to investigate the efficacy.

## Conclusion

Our meta-analysis indicates that QL block is likely to be an effective option for postoperative pain management in patients undergoing hip surgery compared with sham block or no block. The analgesic benefits include the reduced opioid requirement at 24 h and the significantly improved dynamic pain scores in the first 24 h postoperatively in these patients. This study fails to conduct subgroup analysis stratified by the surgery type due to the small number of available studies sometimes. We tend to make cautious conclusions for the effectiveness of QL block on one specific surgery type. Currently, there is very limited evidence comparing QL block with other analgesic techniques for hip surgery. Considering the limited trials available on this topic, further studies with large sample sizes and standardized endpoints should be conducted to evaluate the analgesic effectiveness of QL block.

## Supplementary Information

**Additional file 1.** PRISMA 2020 flow diagram.

**Additional file 2.** PRISMA 2020 checklist.

## Data Availability

The datasets generated and analyzed during the current study are available from the corresponding author on reasonable request.

## Abbreviations

CIs: Confidence intervals
QL: Quadratus lumborum
RCTs: Randomized controlled trials
IME: Intravenous morphine equivalent
LA: Local anesthetic
PONV: Postoperative nausea and vomiting
