# Supplementary material for: Postoperative analgesic effectiveness of quadratus lumborum block: systematic review and meta-analysis for adult patients undergoing hip surgery
Source: J Orthop Surg Res. 2022 May 19;17:282. doi: 10.1186/s13018-022-03172-8 (PMC9118859; doi:10.1186/s13018-022-03172-8)
Supplement: Supplementary file 1 — Additional file 1. PRISMA 2020 flow diagram. [file 13018_2022_3172_MOESM1_ESM.docx]

**Identification of studies via other methods**

**Identification of studies via databases and registers**

Records identified from:

Websites (n=43)

Citation searching (n=13)

Records removed *before screening*:

Duplicate records removed (n=11)

Records marked as ineligible by automation tools (n=33)

Records removed for other reasons (n=35)

Records identified from*:

Databases (n=509)

Registers (n=47)

**Identification**

Records screened

(n=477)

Records excluded**

(n=398)

Reports not retrieved

(n=18)

Reports sought for retrieval

(n=26)

Reports sought for retrieval

(n=79)

Reports not retrieved

(n=59)

**Screening**

Reports excluded:

Paediatric clinical trial (n=4)

Retrospective study (n=3)

Continuous infusion studies

(n=2)

Study with no randomization

(n=1)

Study protocols (n=1)

Reports assessed for eligibility

(n=8)

Reports excluded:

Retrospective study (n=3)

Study protocols (n=1)

Reports assessed for eligibility

(n=20)

Studies included in review

(n=13)

**Included**
